# Royal Jelly Attenuates LPS-Induced Inflammation in BV-2 Microglial Cells through Modulating NF-*κ*B and p38/JNK Signaling Pathways

**DOI:** 10.1155/2018/7834381

**Published:** 2018-04-08

**Authors:** Meng-Meng You, Yi-Fan Chen, Yong-Ming Pan, Yi-Chen Liu, Jue Tu, Kai Wang, Fu-Liang Hu

**Affiliations:** ^1^College of Animal Sciences, Zhejiang University, Hangzhou 310058, China; ^2^Comparative Medical Research Center, Experimental Animal Research Center, Zhejiang Chinese Medical University, Hangzhou 310053, China; ^3^Institute of Apicultural Research, Chinese Academy of Agricultural Sciences, Beijing 100093, China

## Abstract

Royal jelly (RJ), a hive product with versatile pharmacological activities, has been used as a traditional functional food to prevent or treat inflammatory diseases. However, little is known about the anti-inflammatory effect of RJ in microglial cells. The aim of this study is to assess the anti-inflammatory effects of RJ in lipopolysaccharide- (LPS-) induced murine immortalized BV-2 cells and to explore the underlying molecular mechanisms. Our results showed that in LPS-stimulated BV-2 cells, RJ significantly inhibited iNOS and COX-2 expression at mRNA and protein levels. The mRNA expression of IL-6, IL-1*β*, and TNF-*α* was also downregulated by RJ in a concentration-dependent manner. Additionally, RJ protected BV-2 cells against oxidative stress by upregulating heme oxygenase-1 (HO-1) expression and by reducing reactive oxygen species (ROS) and nitric oxide (NO) production. Mechanistically, we found that RJ could alleviate inflammatory response in microglia by suppressing the phosphorylation of I*κ*B*α*, p38, and JNK and by inhibiting the nucleus translocation of NF-*κ*B p65. These findings suggest that RJ might be a promising functional food to delay inflammatory progress by influencing the microglia function.

## 1. Introduction

It is noteworthy that inflammation in the central nervous system (CNS) is associated with the occurrence and progression of some neurodegenerative diseases, including Alzheimer's disease (AD) [1]. Since Eikelenboom and Stam first found the inflammatory mediators in AD brains after 1982, more and more scientists started to explore the relationship between inflammation and AD [2]. At the end of the 1980s, McGeer and Rogers reported that there were lots of activated glia cells in the AD brain [3, 4]. An elevated level of interleukin-18 (IL-18), a detrimental inflammatory cytokine, has also been reported in the brains of AD patients [5]. Since the proposal of the concept “neuroinflammation,” it is now well accepted that one of the most important pathogeneses of AD is chronic long-lasting neuroinflammation [6].

Microglia, a type of resident macrophages located in the brain, is the major homeostasis regulator of CNS [7]. Under normal physiological conditions, microglia can upregulate anti-inflammatory mediators and phagocytose the toxic cellular debris with high efficiency. In spite of this, the neuron growth factor (NGF) secreted by microglia is a trophic factor for neurons [8, 9]. However, microglia will switch to an activated phenotype (M1 type) and generate highly detrimental mediators when their microenvironment becomes stressful. All of these neurotoxic mediators will do harm to neurons and glial cells, and the brain homeostasis will be disturbed, which is the main cause of most neurodegenerative diseases [10]. Due to the dual effects of microglia, appropriate activation of microglia is needed to keep the brain away from endogenous or exogenous toxins. It was reported that lipopolysaccharide (LPS), a bacterial endotoxin, could stimulate microglia to M1 type by combining with its receptors and activating inflammatory and oxidative pathways [11]. Systemic administration of LPS is known to have an influence on neurobiological conditions, promoting the production of inflammatory factors [12]. In studies with cell cultures, inflammation of BV-2 cell line and primary microglia is frequently modeled by LPS [13–15]. Therefore, in this study, we employed LPS as stimuli to induce inflammatory damage in BV-2 cells. The production of proinflammatory and cytotoxic mediators by microglia, such as IL-6, nitric oxide (NO), reactive oxygen species (ROS), inducible nitric oxide synthase (iNOS), IL-1*β*, cyclooxygenase-2 (COX-2), and tumor necrosis factor-*α* (TNF-*α*), will be significantly elevated after LPS treatment [16]. In addition, mounting evidence has shown that LPS can cause inflammation via regulating the activity of nuclear factor kappa B (NF-*κ*B), mitogen-activated protein kinases (MAPK), signal transducer and activator of transcription 1 (STAT1), and activator protein (AP-1) [17–19].

To improve neurodegenerative disorders, diverse treatment modalities were adopted to reduce inflammation caused by microglia. Nonsteroidal anti-inflammatory drugs (NSAIDs) are a class of drugs used to treat inflammation, mild to moderate pain, and fever, block the COX enzymes, and reduce the production of prostaglandins throughout the body [20]. Some studies have already reported that long-term administration of NSAIDs was capable of attenuating neuroinflammation and improving tau pathology in transgenic AD mice [21]. However, NSAIDs may increase the risk of developing gastrointestinal and cardiovascular diseases, which greatly limits their range of application [22, 23]. Nowadays, a growing number of studies have focused on the antineuroinflammatory effects of natural functional foods [24, 25]. Royal jelly (RJ), a bee product produced by the hypopharyngeal and mandibular glands of worker bees, is chemically composed of water, proteins, carbohydrates, vitamins, lipids, and other bioactive compounds [26]. As a functional food, RJ has various pharmacological activities, including anti-inflammatory [27], antihypertensive [28], antioxidative [29], and memory function restoration activities [30]. However, whether RJ could protect microglial cells from inflammatory stimuli remains elusive. Here, the aim of this study is to assess the influence of RJ on the function of BV-2 cells under LPS-induced inflammatory conditions *in vitro* and to further explore the underlying mechanisms.

## 2. Materials and Methods

### 2.1. Chemicals and Reagents

RJ was purchased from Fengzhiyu Apicultural Co. Ltd. (Hangzhou, China). The purity of RJ is 100%, and its composition is in line with international standards (ISO12824: 2016). RJ was suspended in sterile phosphate-buffered saline (PBS) at concentration of 20 mg/mL, and RJ stock solution was stored at −20°C until use. LPS (*Escherichia coli* O111: B4), 2′,7′-dichlorofluorescein diacetate (DCFH-DA) and alkaline phosphatase-conjugated antibody (anti-rabbit IgG) were purchased from Sigma (St. Louis, MO, USA). Fetal bovine serum (FBS) was purchased from Gibco BRL (Grand Island, NY, USA). Cell counting kit-8 was purchased from Dojindo (Japan). Griess reagent, NaNO_2_, and 4′6-diamidino-2-phenylindole (DAPI) were purchased from Sangon Biotechnology, Co. Ltd. (Shanghai, China). ELISA kits for IL-6 and TNF-*α* were purchased from Neobioscience (Shanghai, China). PrimeScript RT Master Mix real-time kits were purchased from Takara (Dalian, China). Primary antibodies against NF-*κ*B p65, I*κ*B*α*, COX-2, and iNOS were purchased from Abcam (Cambridge, Massachusetts, USA). Primary antibodies against p-I*κ*B*α*, JNK, p-JNK, p38, p-p38, ERK, and p-ERK were purchased from Cell Signaling Technology (Danvers, MA, USA).

### 2.2. Cell Culture

The murine microglial cell line BV-2 was a generous gift from Professor Yunbi Lu (School of Medicine, Zhejiang University, China). BV-2 cells were cultured in high-glucose DMEM supplemented with 10% FBS, 2 mM glutamine, 100 U/mL penicillin, and 100 *μ*g/mL streptomycin at 37°C in a 5% CO_2_ atmosphere. After reaching 80%–90% confluency, BV-2 cells were seeded into plates for 24 h incubation in high-glucose DMEM supplemented with 10% FBS. The cell culture medium was then removed and washed 3 times with PBS. After that, RJ stock solution was diluted into desired concentrations using serum-free medium, and the cells were pretreated with a mixture of RJ and serum-free medium for 1 h followed by addition of LPS (1 *μ*g/mL). The incubation period of LPS varies depending on the experiment.

### 2.3. Cell Viability Assay

The neurotoxicity effect of RJ was measured by cell counting kit-8 assay according to the manufacturer's instruction. BV-2 cells were seeded into 96-well plates for 24 h and were then incubated with different concentrations of RJ (0, 0.3, 1, 3, and 6 mg/mL) for another 24 h. Following that, the cells were incubated with 10 *μ*L cell counting kit-8 solution for 2 h at 37°C. The percentage of surviving cells was measured using microplate reader (Bio-Rad, Model 550, CA) at 450 nm.

### 2.4. Determination of NO Production

The amount of nitrite accumulation in the culture medium of BV-2 cells was measured using Griess reagent. BV-2 cells were plated in 24-well plates for 24 h. After that, cells were treated with RJ (0, 0.3, 1, and 3 mg/mL) for 1 h prior to 24 h LPS (1 *μ*g/mL) stimulation. After the collection of culture medium, the supernatant absorbance was determined at 540 nm using a microplate absorbance reader. Nitrite concentrations were calculated according to nitrite standard curve.

### 2.5. Reactive Oxygen Species Detection

The ROS assay in BV-2 cells was carried out as described before with some minor modifications [31]. Cells were seeded into 12-well plates for 24 h incubation, and then various concentrations of RJ (0, 0.3, 1, and 3 mg/mL) were added to the plates for 1 h followed by LPS (1 *μ*g/mL) treatment. After 24 h incubation, 10 *μ*M (final concentration) DCFH-DA (a sensitive fluorescent probe) was added to each well for 30 min at 37°C. After that, the cells were detached with trypsin and washed with PBS for three times by centrifugation. The production of ROS was measured by BD FACSCalibur flow cytometer at emission and excitation wavelengths of 535 and 488 nm.

### 2.6. RNA Extraction and Quantitative Real-Time PCR

In order to measure the mRNA expression of inflammatory mediators in BV-2 microglial cells, total RNA from BV-2 cells was isolated using RNA extraction kits (Aidlab Biotechnologies Co. Ltd., Beijing, China) and was quantified by NanoDrop spectrophotometer (ND-2000, NanoDrop Technologies, USA). For reverse transcription, 500 nanograms of total RNA of each sample was reverse transcribed into cDNA using PrimeScript RT Master Mix (TaKaRa, Dalian, China). After cDNA synthesis, quantitative real-time PCR was conducted using a Master cycle reprealplex (Eppendorf, Hamburg, Germany) with a SYBR Premix Ex Taq (TaKaRa, Dalian, China) via a standard two-step PCR reaction according to the manufacturer's instruction. The mRNA expression of IL-6, iNOS, COX-2, TNF-*α*, heme oxygenase-1 (HO-1), monocyte chemotactic protein 1 (MCP-1), and IL-1*β* was normalized to GAPDH. The primer sequences used in this study are listed in Table 1.

### 2.7. Cytokine Assay (ELISA Assay)

The protein levels of IL-6 and TNF-*α* in culture medium were quantified by enzyme-linked immunosorbent assay (ELISA) kits. BV-2 cells were pretreated with RJ (0.3, 1, and 3 mg/mL) for 1 h and were then exposed to LPS (1 *μ*g/mL) for 24 h. Cell-free supernatants were collected and tested according to manufacturer's protocols. Briefly, supernatants were added to microplate wells, which were coated with specific antibodies in advance. After 1.5 h of incubation at 37°C, unbound substances were washed away, and enzyme-linked antibody was added to each well followed by 1 h incubation at 37°C. Then, the second wash was carried out to remove unbound antibody-enzyme reagents. Finally, the substrate solution was pipetted into each well for 15 min, and the stop solution was added to end the color development. Absorbance at 450 nm was measured using a microplate reader.

### 2.8. Immunofluorescence Staining

For the detection of NF-*κ*B nuclear translocation, BV-2 cells were pretreated with RJ (0.3, 1, and 3 mg/mL) for 1 h and were subsequently incubated by 1 *μ*g/mL LPS for 45 min. After that, BV-2 cells were fixed with precooled 4% paraformaldehyde, permeabilized with 0.3% Triton X-100, and blocked with 5% bovine serum albumin (BSA) for 30 min, respectively. Then, the monoclonal primary antibody to NF-*κ*B p65 (1 : 250) was applied overnight at 4°C, followed by incubation with Alexa Fluor 488-conjugated goat anti-rabbit IgG (1 : 250) for 1 h at 37°C in dark. At last, BV-2 cells were stained with DAPI for 5 min at room temperature, and after being washed three times with PBS, the cells were examined under a confocal laser microscope (Leica, TCS SP5, Germany).

### 2.9. Cellular Protein Extraction and Western Blot Analysis

The Western blot analysis was conducted according to protocols described previously [32]. Briefly, after different treatments, BV-2 cells were washed with precooled PBS twice, and cytoplasmic proteins were lysed with NP-40 which has already been added with a protease/phosphatase inhibitor cocktail (Roche, Basel, Switzerland) in advance. The cells were then collected, and bicinchoninic acid (BCA) assay was conducted to assess the protein concentration using BCA protein assay kit (Weiao Biotechnology, Shanghai, China). Degenerated proteins (30 *μ*g) were separated by 12% sodium dodecyl sulphate-polyacrylamide gel electrophoresis (SDS-PAGE) and were then transferred onto polyvinylidene difluoride (PVDF) membranes (Millipore, Billerica, MA). PVDF membranes were blocked with 5% skim milk at room temperature for 1 h to avoid nonspecific binding, and immunoblots were incubated for overnight at 4°C with primary antibodies that specifically detect I*κ*B*α*, p-I*κ*B*α*, JNK, p-JNK, p38, p-p38, ERK, p-ERK, COX-2, iNOS, and *β*-tubulin. After that, horseradish peroxidase-conjugated secondary antibodies were incubated for 1 h at room temperature. Blots were developed by NBT/BCIP, and band intensities were quantified using Quantity One Software.

### 2.10. Statistical Analysis

The data are expressed as means ± SEM from three independent experiments. One-way ANOVA with post hoc Tukey's test was used to determine statistical difference, and *P* values < 0.05 were considered statistically significant. Statistical analyses were performed using GraphPad Prism 6.0 (GraphPad Software Inc., La Jolla, CA, USA).

## 3. Results

### 3.1. Effect of RJ on BV-2 Cell Viability

**Figure 1:**
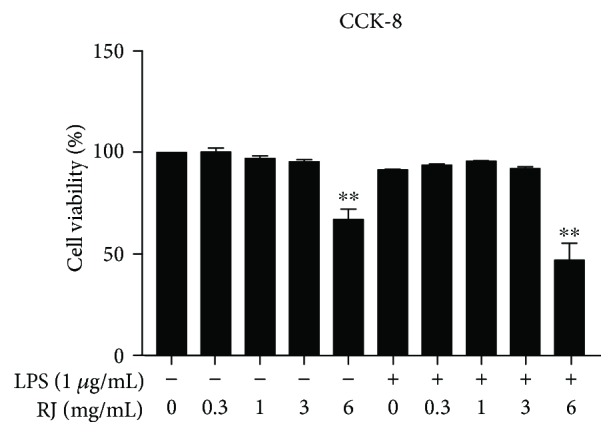
Cell viability of RJ-treated microglia was determined by cell counting kit-8 assay. BV-2 cells were treated with 0, 0.3, 1, 3, and 6 mg/mL RJ for 24 h, respectively, and the results are expressed as proportions of surviving cells compared with controls. Data are presented as means ± SEM, and group differences were analyzed by one-way ANOVA with post hoc Tukey's test. ^∗∗^ *P* < 0.01 compared with untreated control group.

To determine the appropriate concentrations of RJ treatments, we carried out the cell counting kit-8 assay to measure the viability of cells treated by RJ alone and cells cotreated with RJ/LPS (Figure 1). Based on our cell viability histogram, treatments of RJ up to 3 mg/mL for 24 h had no cytotoxic effects in comparison with the control group. However, RJ at a dose of 6 mg/mL significantly reduced the viability of BV-2 cells either by itself or in combination with LPS (*P* < 0.01). According to these results, we chose RJ at a concentration of 0.3, 1, and 3 mg/mL in the following studies.

### 3.2. Effects of RJ on LPS-Induced Production of NO and ROS and Protein Expression of iNOS and COX-2 in BV-2 Cells

**Figure 2:**
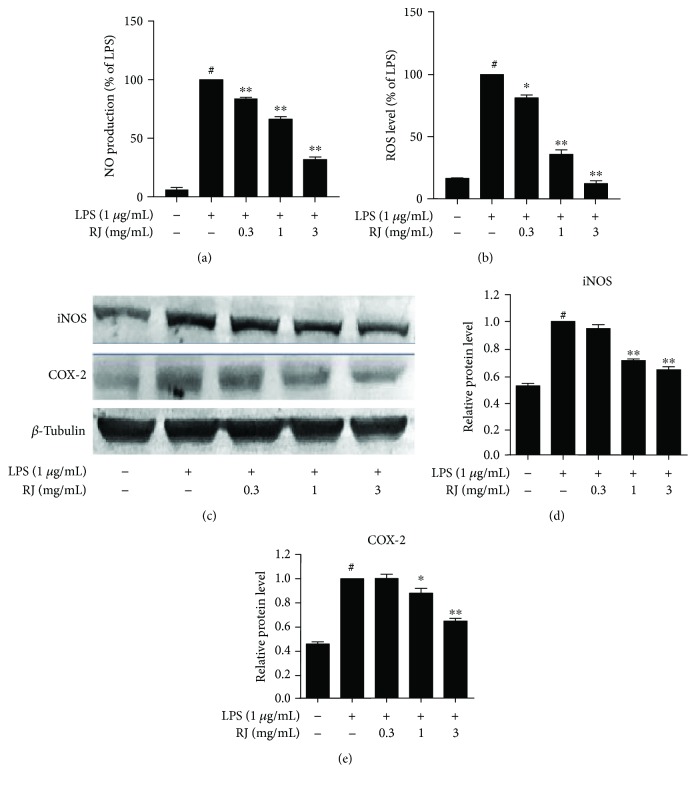
Effects of RJ on LPS-induced production of NO and ROS, as well as the protein expression of iNOS and COX-2 in BV-2 cells. BV-2 cells were pretreated with RJ (0, 0.3, 1, and 3 mg/mL) for 1 h, followed by 24 hours of incubation with LPS (1 *μ*g/mL). The NO production in cell supernatants was detected by Griess reaction (a), and intracellular ROS levels were measured using DCF fluorescence as described in the text (b). BV-2 cells were pretreated in the same way as described above and were then stimulated with 1 *μ*g/mL LPS for 24 h. Proteins were extracted, and Western blot analysis was conducted using specific antibodies against iNOS and COX-2 (c–e). *β*-Tubulin protein was used here as an internal control. Data are presented as means ± SEM, and group differences were analyzed by one-way ANOVA with post hoc Tukey's test. ^#^ *P* < 0.05 compared with untreated control group; ^∗^ *P* < 0.05, ^∗∗^ *P* < 0.01 compared with the group treated with LPS alone.

NO levels in cell culture medium were markedly elevated after 24 h of LPS treatment compared to the control group, whereas RJ significantly lowered this level at all three concentrations (*P* < 0.01) (Figure 2(a)). At 3 mg/mL of RJ, NO production was suppressed by more than 60% compared to the LPS treatment group. In addition, fluorescence-based ROS assay was carried out to assess the ROS production by BV-2 cells (Figure 2(b)). We found that preincubation of RJ for 1 h could suppress the increase of ROS levels caused by LPS in a dose-dependent manner. Western blot was used to assess the protein expression of iNOS and COX-2. As shown in Figures 2(c)–2(e), LPS treatment for 24 h evidently promoted the expression of iNOS and COX-2, while RJ pretreatment (1 mg/mL and 3 mg/mL) markedly suppressed these increases. However, RJ at a low concentration (0.3 mg/mL) did not work in this case.

### 3.3. Effects of RJ on LPS-Induced mRNA Expression of Inflammatory Mediators

**Figure 3:**
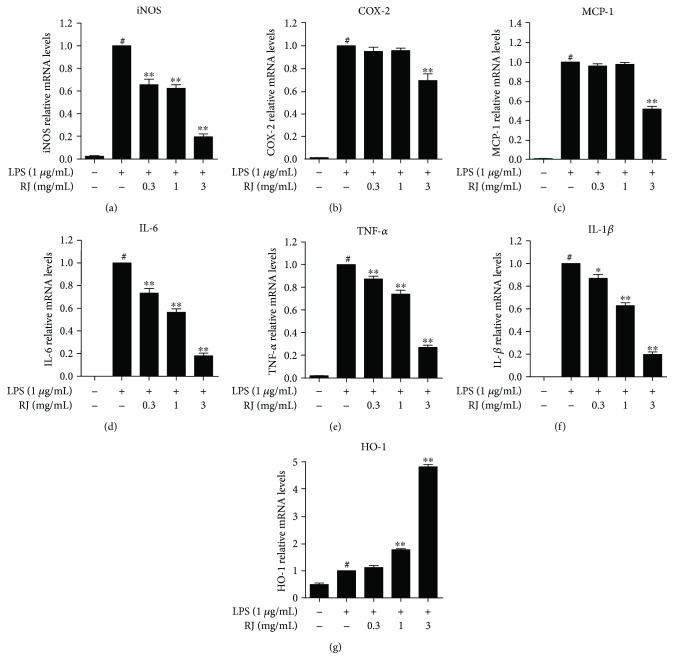
Effects of RJ on LPS-induced mRNA expression of inflammatory mediators in BV-2 microglial cells. BV-2 cells were preincubated in the culture medium with RJ (0, 0.3, 1, and 3 mg/mL) for 1 h and were then stimulated with 1 *μ*g/mL LPS for another 6 h. BV-2 cells were harvested for RNA extraction with Trizol, and the mRNA expressions of iNOS (a), COX-2 (b), MCP-1 (c), IL-6 (d), TNF-*α* (e), IL-1*β* (f), and HO-1 (g) were measured by qRT-PCR. Data are presented as means ± SEM, and group differences were analyzed by one-way ANOVA with post hoc Tukey's test. ^#^ *P* < 0.05 compared with untreated control group; ^∗^ *P* < 0.05, ^∗∗^ *P* < 0.01 compared with the group treated with LPS alone.

The effect of RJ on LPS-induced mRNA expression of iNOS, COX-2, IL-6, IL-1*β*, TNF-*α*, MCP-1, and HO-1 was analyzed by qRT-PCR (Figure 3). Our results showed that COX-2, iNOS, and MCP-1 mRNA expressions were significantly elevated in response to LPS induction for 6 h (*P* < 0.01). In contrast, 1 h pretreatment of 3 mg/mL RJ markedly lowered these expressions (*P* < 0.01), though RJ did not downregulate the expression of COX-2 and MCP-1 at a concentration lower than 3 mg/mL (Figures 3(a)–3(c)). Regarding IL-6, IL-1*β*, and TNF-*α*, pretreatment with RJ for 1 h caused a concentration-dependent decrease of mRNA expression in BV-2 cells (Figures 3(d)–3(f)). It was reported recently that HO-1 could act as a target for treating neurodegenerative diseases [33]. The qRT-PCR results showed that RJ could upregulate the expression of HO-1 significantly, whereas HO-1 expression in BV-2 cells pretreated by 3 mg/mL RJ before LPS stimulation was about 8 times higher than that of the control group. This demonstrates that RJ pretreatment could effectively inhibit the expression of key inflammatory mediators in LPS-stimulated BV-2 cells.

### 3.4. Effects of RJ on LPS-Induced Cytokine Production

**Figure 4:**
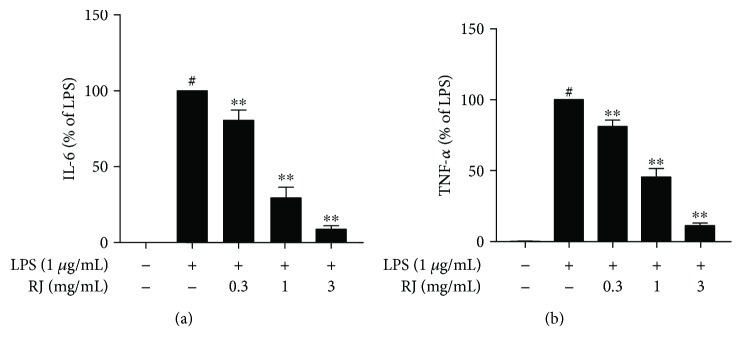
Effects of RJ on LPS-induced cytokine production. BV-2 cells were pretreated with RJ (0, 0.3, 1, and 3 mg/mL) for 1 h, followed by a 24 h incubation with 1 *μ*g/mL LPS. IL-6 (a) and TNF-*α* (b) released into the cell culture medium were quantified by ELISA kits. Data are presented as means ± SEM, and group differences were analyzed by one-way ANOVA with post hoc Tukey's test. ^#^ *P* < 0.05 compared with untreated control group; ^∗∗^ *P* < 0.01 compared with the group treated with LPS alone.

As shown in Figure 4, the production of proinflammatory cytokines such as IL-6 and TNF-*α* was strongly upregulated after 24 h treatment of LPS compared to the control group. On the other hand, RJ pretreatment inhibited this increase in a dose-dependent manner, indicating that RJ could work efficiently to suppress the secretion of inflammatory mediators in LPS-induced BV-2 cells.

### 3.5. Effects of RJ on LPS-Induced Activation of NF-*κ*B and p38/JNK Pathways

**Figure 5:**
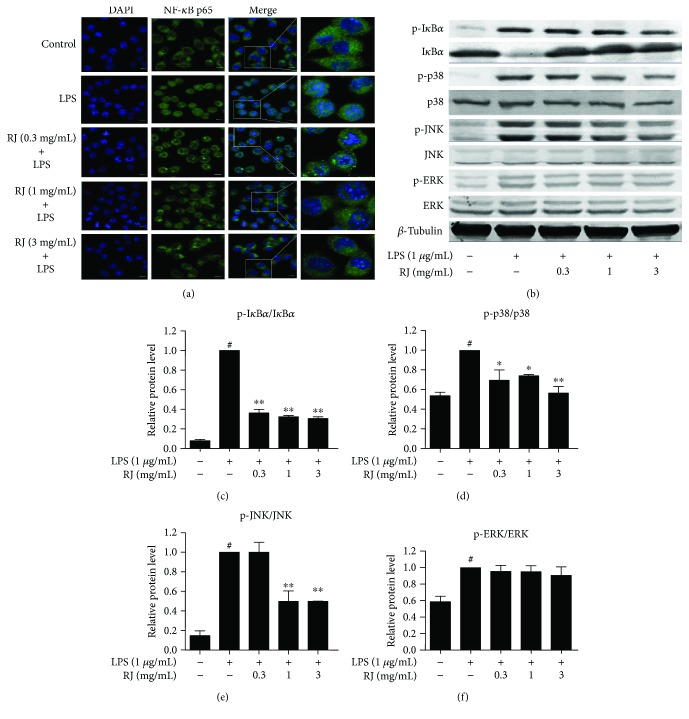
Effects of RJ on LPS-induced activation of NF-*κ*B and MAPK pathways. BV-2 cells were pretreated with RJ (0, 0.3, 1, and 3 mg/mL) for 1 h, followed by a 45 min incubation with 1 *μ*g/mL LPS. NF-*κ*B p65 nucleus translocation was observed by confocal laser microscope, scale bar = 10 *μ*m (a). BV-2 cells were pretreated in the same way as described above. Proteins were extracted, and Western blot analysis was conducted using specific antibodies (b–f). *β*-Tubulin protein was used as an internal control. Data are presented as means ± SEM, and group differences were analyzed by one-way ANOVA with post hoc Tukey's test. ^#^ *P* < 0.05 compared with untreated control group; ^∗^ *P* < 0.05, ^∗∗^ *P* < 0.01 compared with the group treated with LPS alone.

Our results showed that the phosphorylation levels of I*κ*B*α*, p38, c-Jun NH2-terminal kinases (JNK), and extracellular signal-regulated kinases (ERK) peaked at 45 minutes after the LPS stimulation (Supplementary Figure 1). Thus, we chose 45 min as the LPS stimulation period. When NF-*κ*B is activated, p65 subunit of NF-*κ*B complex will translocate from cytoplasm to nucleus. From our immunocytochemistry results, it was evident that GFP-labeled NF-*κ*B p65 submit was mainly located in cytoplasm under normal physiological conditions, and it translocated to nucleus when BV-2 cells were activated by 1 *μ*g/mL LPS. However, RJ inhibited NF-*κ*B p65 translocation under LPS exposure (Figure 5(a)). In addition, Western blot results revealed that LPS treatment (1 *μ*g/mL) significantly promoted the I*κ*B*α* degradation, which was consistent with previous studies [34, 35]. RJ at all three concentrations could significantly inhibit the degradation of I*κ*B*α* and decrease the phosphorylation levels of I*κ*B*α* and p38 elevated by LPS treatment (Figures 5(b)–5(d)). Although RJ at 0.3 mg/mL did not inhibit the activation of JNK pathway, 1 mg/mL and 3 mg/mL RJ markedly lowered the protein expression of p-JNK (Figures 5(b) and 5(e)). The LPS-induced phosphorylation of p38 and JNK was also strongly inhibited by their inhibitors SB203580 and SP600125 (Supplementary Figure 2). These results suggest that RJ reduces the inflammatory response of BV-2 microglia to LPS stimulation by inhibiting the phosphorylation of I*κ*B*α*, p38, and JNK. The phosphorylation level of ERK was significantly increased after LPS treatment for 45 min; however, RJ at all three concentrations do not have any effect on p-ERK level (Figures 5(b) and 5(f)). We speculate that ERK pathway may not be involved in the mechanism by which RJ exerts the anti-inflammatory effects in BV-2 cells.

## 4. Discussion

It is widely accepted that CNS inflammation in neurodegenerative diseases is a double-edged sword. It acts as an innate defense mechanism against various stimuli and removes toxic as well as other detrimental substances. However, it can also promote the neurodegenerative process if the inflammation is sufficiently severe [36]. Some studies have already verified that anti-inflammatory drugs, such as ibuprofen, could alleviate CNS inflammation and inhibit the activation of microglial cells [37, 38]. Therefore, it is of great interest to investigate whether RJ, a natural functional food with considerable anti-inflammatory activities, could alleviate inflammation damage caused by microglial cells. In the present study, we used LPS at 1 *μ*g/mL to treat BV-2 cells because our preliminary experimental results showed that 1 *μ*g/mL of LPS could significantly cause inflammation without affecting cell viability (data not shown). In addition, previous studies have also used 1 *μ*g/mL of LPS (*Escherichia coli* O111: B4) as inflammatory stimuli in BV-2 microglial cells [39]. We tested the anti-inflammatory effects of RJ in LPS-treated BV-2 cells and provided firsthand evidence that RJ could significantly reduce the inflammatory impact of LPS *in vitro*. Additionally, we demonstrated that RJ displayed excellent anti-inflammatory effects in BV-2 microglial cells via NF-*κ*B, JNK, and p38 signaling pathways.

Microglia, primary immune cells in the brain, are reported to enhance neuroinflammation in most neurodegenerative diseases [40]. Previous study has reported that, in the case of LPS stimulation, BV-2 cells have a similar gene profiling and cytokine secretion functions compared with primary microglia [41]. In addition, another study showed that LPS caused similar effects in BV-2 cells and primary microglia regarding the COX-2 mRNA and protein expression [42]. In this study, we used the BV-2 microglia cell line to assess the effects of RJ on microglia. IL-1*β*, IL-6, and TNF-*α* are three major proinflammatory cytokines during the inflammation process; in our study, RJ inhibited the transcription of TNF-*α*, IL-1*β*, and IL-6 in a dose-dependent manner, demonstrating the potential of RJ to protect BV-2 cells against LPS-induced inflammation. In spite of this, our ELISA results, consistent with the qRT-PCR assay, showed that RJ treatment under inflammatory conditions also blocked the secretion of proinflammatory mediators, indicating that RJ suppressed the synthesis and release of proinflammatory mediators in BV-2 cells at both transcriptional and translational levels. COX-2 is the rate-limiting enzyme for converting arachidonic acid to proinflammatory prostaglandins, and it plays a pivotal role in chronic and acute inflammation [43]. Our Western blot results indicated that RJ could inhibit COX-2 protein expression in a dose-dependent manner. We also investigated the gene expression of COX-2, the results from which showed that RJ could downregulate the mRNA expression of COX-2, but only 3 mg/mL of RJ caused a significant decrease. One explanation is that RJ at low concentrations could inhibit the production of COX-2 at translational level, but not at transcriptional level, which means that RJ at different concentrations will participate in different biological processes. In addition, the effective concentration for RJ to inhibit COX-2 mRNA expression may be relatively higher than that of other mediators, that is, IL-6, IL-1*β*, and TNF-*α*. We used Griess reagent to assess the secretion of NO by BV-2 cells in this study, and the results showed that LPS significantly increased NO production, and RJ at all three different concentrations reversed this situation, illustrating that RJ could improve inflammation caused by excessive NO and that it has a broad therapeutic window against neuroinflammation. LPS treatment in BV-2 cells also caused oxidative stress by increasing ROS levels [44]. Furthermore, the production of ROS could oxidize proteins, lipids, and DNA, and it is considered to be a vital mediator in the AD process [45]. We observed that RJ pretreatment showed significant protective effects in BV-2 cells by scavenging ROS compared to the LPS-treated group, meaning that RJ could work well to maintain cellular redox balance and may inhibit the onset or progress of some oxidative-related diseases, such as neurodegenerative diseases.

It was reported that LPS stimulation induces inflammation by activating NF-*κ*B and MAPK pathways [46, 47]. Several agents have been previously shown to exert anti-inflammatory effects by inhibiting MAPK activation and DNA binding activity of NF-*κ*B both in RAW 264.7 cells and in microglial BV-2 cells [48, 49]. In the present study, we explored the signaling-based mechanisms of the anti-inflammatory effects of RJ in microglial cells. First, we used LPS at 1 *μ*g/mL to simulate BV-2 cells and observed that the phosphorylation levels of I*κ*B*α*, JNK, p38, and ERK peaked at 45 min. However, Jayasooriya et al. reported that the phosphorylation levels of I*κ*B*α* peaked at 30 min after LPS stimulation in their studies [50]. We speculated that this divergence may be ascribed to different LPS stimulus concentrations or different lineages of BV-2 cells used in our study. Our immunofluorescence assays and Western blot results demonstrated that RJ inhibited the production of inflammatory mediators through NF-*κ*B, p38, and JNK pathways. Although LPS significantly activated ERK pathway, it was not suppressed by RJ. According to previous studies, *trans*-10-hydroxy-2-decenoic acid (10-HDA), the major lipid component of RJ, exerts anti-inflammatory effect via the inhibition of LPS-induced NF-*κ*B activation observed in the murine macrophage cell line RAW 264 [51]. In addition, 10-HDA could also alleviate inflammation in rheumatoid arthritis synovial fibroblasts through blocking p38 and JNK pathways, but had no effect on ERK activity [52], which was consistent with our Western blot results. Thus, one hypothesis is that 10-HDA is an active component of RJ's anti-inflammatory effect in microglial cells.

HO-1, an antioxidant gene, is related to immunologic defense in immune cells, including BV-2 microglia cells [53]. Previous studies have reported that various compounds exert the anti-inflammatory activity in microglial cells not only through inhibiting NF-*κ*B pathway, but also through activating Nrf2 pathway and antioxidant response element (ARE), as well as through increasing the synthesis of some antioxidant enzymes, including HO-1 [54, 55]. RJ, according to our results, strikingly upregulated the mRNA expression of HO-1 and also exerted anti-inflammatory effects via NF-*κ*B pathway. Thus, we speculate that the anti-inflammatory effects of RJ may be attributed, at least in part, to the regulatory effects of HO-1. In addition, it would be of great significance to explore whether there is an interreaction between NF-*κ*B pathway and Nrf-2 pathway in the next study. In addition, further studies are required to determine the antineuroinflammatory effects of RJ in animal models.

## 5. Conclusion

In this study, we firstly assessed the anti-inflammatory effects of RJ on LPS-stimulated BV-2 cells and further explored the underlying mechanisms. Our results demonstrated that RJ contributed to suppressing inflammatory damage caused by microglial cells and suggested RJ as a promising functional food for delaying inflammatory progress in the future.

## Figures and Tables

**Table 1:** Primer sequence used in qRT-PCR.

Gene	Forward primer	Reverse primer	Product size (bp)	GenBank accession number
IL-6	5′-CTCTGCAAGAGACTTCCATCC-3′	5′-GAATTGCCATTGCACAACTC-3′	210	NM_031168.1
iNOS	5′-TTTCCAGAAGCAGAATGTGACC-3′	5′-AACACCACTTTCACCAAGACTC-3′	294	NM_010927.3
COX-2	5′-GAAATATCAGGTCATTGGTGGAG-3′	5′-GTTTGGAATAGTTGCTCATCAC-3′	237	NM_011198.3
TNF-*α*	5′-CCACGCTCTTCTGTCTACTG-3′	5′-ACTTGGTGGTTTGCTACGAC-3′	169	NM_013693.2
HO-1	5′-ACATTGAGCTGTTTGAGGAG-3′	5′-TACATGGCATAAATTCCCACTG-3′	241	NM_010442.2
MCP-1	5′-AAGAAGCTGTAGTTTTTGTCACCA-3′	5′-TGAAGACCTTAGGGCAGATGC-3′	155	NM_011333.3
IL-1*β*	5′-ATCTCGCAGCAGCACATCAAC-3′	5′-TGTTCATCTCGGAGCCTGTAGT-3′	239	NM_008361.3
GAPDH	5′-GAGAAACCTGCCAAGTATGATGAC-3′	5′-TAGCCGTATTCATTGTCATACCAG-3′	212	NM_008084.2

## Abbreviations

CNS: Central nervous system
AD: Alzheimer's disease
IL: Interleukin
NGF: Neuron growth factor
LPS: Lipopolysaccharide
NO: Nitric oxide
ROS: Reactive oxygen species
iNOS: Inducible nitric oxide synthase
COX-2: Cyclooxygenase-2
TNF-*α*: Tumor necrosis factor-*α*
NF-*κ*B: Nuclear factor kappa B
MAPK: Mitogen-activated protein kinases
STAT1: Signal transducer and activator of transcription 1
AP-1: Activator protein
NSAIDs: Nonsteroidal anti-inflammatory drugs
RJ: Royal jelly
HO-1: Heme oxygenase-1
MCP-1: Monocyte chemotactic protein 1
JNK: c-Jun NH2-terminal kinases
ERK: Extracellular signal-regulated kinases
10-HDA: *trans*-10-hydroxy-2-decenoic acid
ARE: Antioxidant response element.

## Supplementary Materials

Supplementary Materials Figure 1: effects of RJ on the phosphorylation levels of I*κ*B*α*, ERK, p38, and JNK in LPS-stimulated BV-2 cells at different time points (A–E). Data are presented as means ± SEM, and group differences were analyzed by one-way ANOVA with post hoc Tukey's test. ^#^ *P* < 0.05 compared with untreated control group; ^∗^ *P* < 0.05, ^∗∗^ *P* < 0.01 compared with the group treated with LPS alone. Figure 2: effects of JNK inhibitor (SP600125) and p38 inhibitor (SB203580) on the phosphorylation levels of JNK and p38 in LPS-stimulated BV-2 cells. BV-2 cells were pretreated with or without RJ (3 mg/mL), SP600125 (20 *μ*M), or SB203580 (10 *μ*M) for 1 h, respectively, then treated with or without LPS for 45 min. The phosphorylated levels of JNK (A) and p38 (B) were detected by Western blot analysis. *β*-Tubulin protein was used as an internal control. Data are presented as means ± SEM, and group differences were analyzed by one-way ANOVA with post hoc Tukey's test. ^#^ *P* < 0.05 compared with untreated control group; ^∗^ *P* < 0.05, ^∗∗^ *P* < 0.01 compared with the group treated with LPS alone.
