# Supplementary material for: Royal Jelly Attenuates LPS-Induced Inflammation in BV-2 Microglial Cells through Modulating NF-κB and p38/JNK Signaling Pathways
Source: Mediators Inflamm. 2018 Apr 8;2018:7834381. doi: 10.1155/2018/7834381 (PMC5911318; doi:10.1155/2018/7834381)
Supplement: Supplementary Materials — Figure 1: effects of RJ on the phosphorylation levels of IκBα, ERK, p38, and JNK in LPS-stimulated BV-2 cells at different time points (A–E). Data are presented as means ± SEM, and group differences were analyzed by one-way ANOVA with post hoc Tukey's test. # P < 0.05 compared with untreated control group; ∗ P < 0.05, ∗∗ P < 0.01 compared with the group treated with LPS alone. Figure 2: effects of JNK inhibitor (SP600125) and p38 inhibitor (SB203580) on the phosphorylation levels of JNK and p38 in LPS-stimulated BV-2 cells. BV-2 cells were pretreated with or without RJ (3 mg/mL), SP600125 (20 μM), or SB203580 (10 μM) for 1 h, respectively, then treated with or without LPS for 45 min. The phosphorylated levels of JNK (A) and p38 (B) were detected by Western blot analysis. β-Tubulin protein was used as an internal control. Data are presented as means ± SEM, and group differences were analyzed by one-way ANOVA with post hoc Tukey's test. # P < 0.05 compared with untreated control group; ∗ P < 0.05, ∗∗ P < 0.01 compared with the group treated with LPS alone. [file 7834381.f1.pdf]

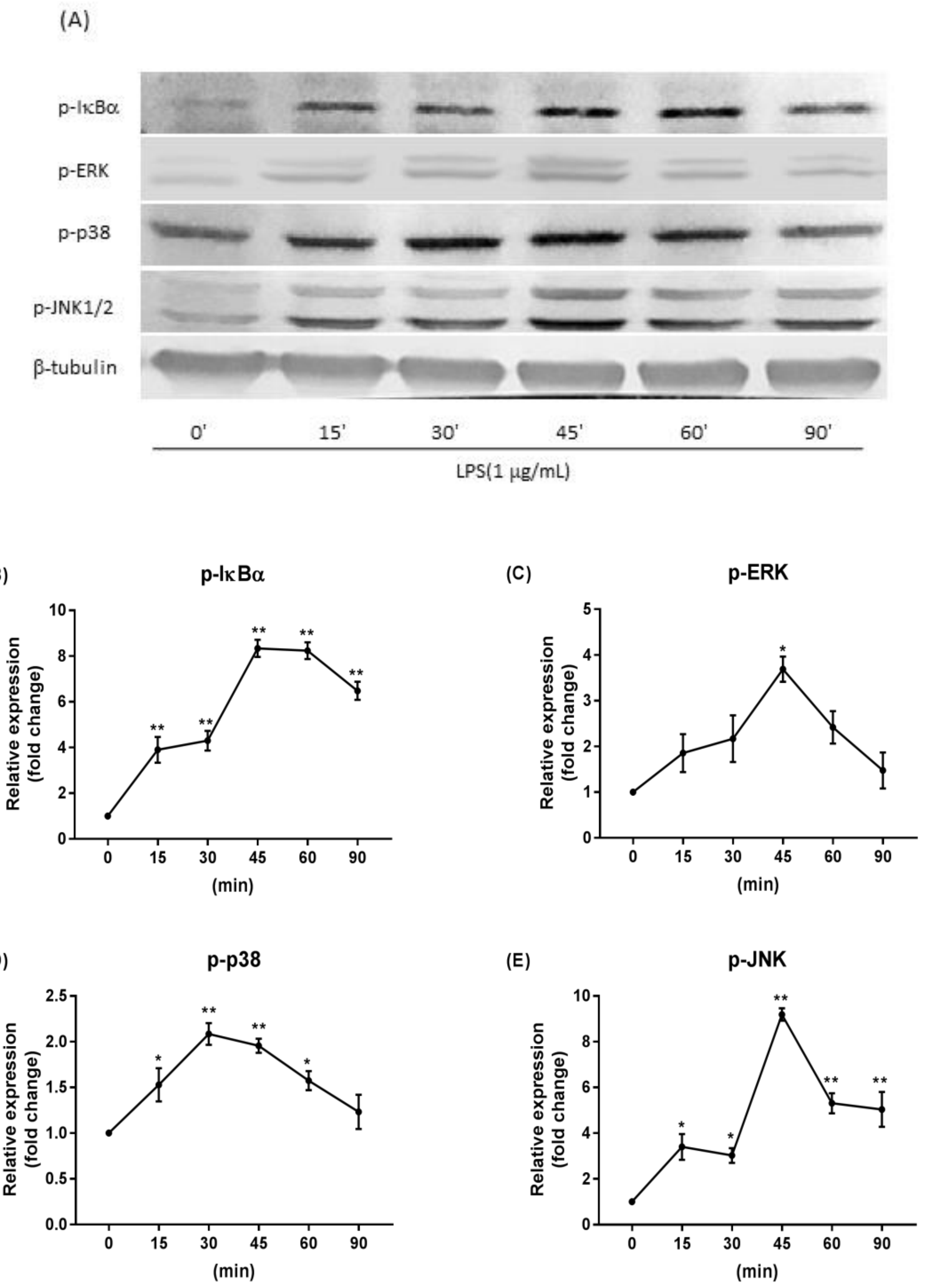

FIGURE 1: Effects of RJ on the phosphorylation levels of IκBα, ERK, p38 and JNK in LPS-stimulated BV-2 cells at different time points (A-E). Data are presented as means ± SEM, and group differences were analyzed by one-way ANOVA with *post hoc* Tukey's test. $^{\#}P<0.05$ compared with untreated control group; *$P<0.05$, **$P<0.01$ compared with the group treated with LPS alone.

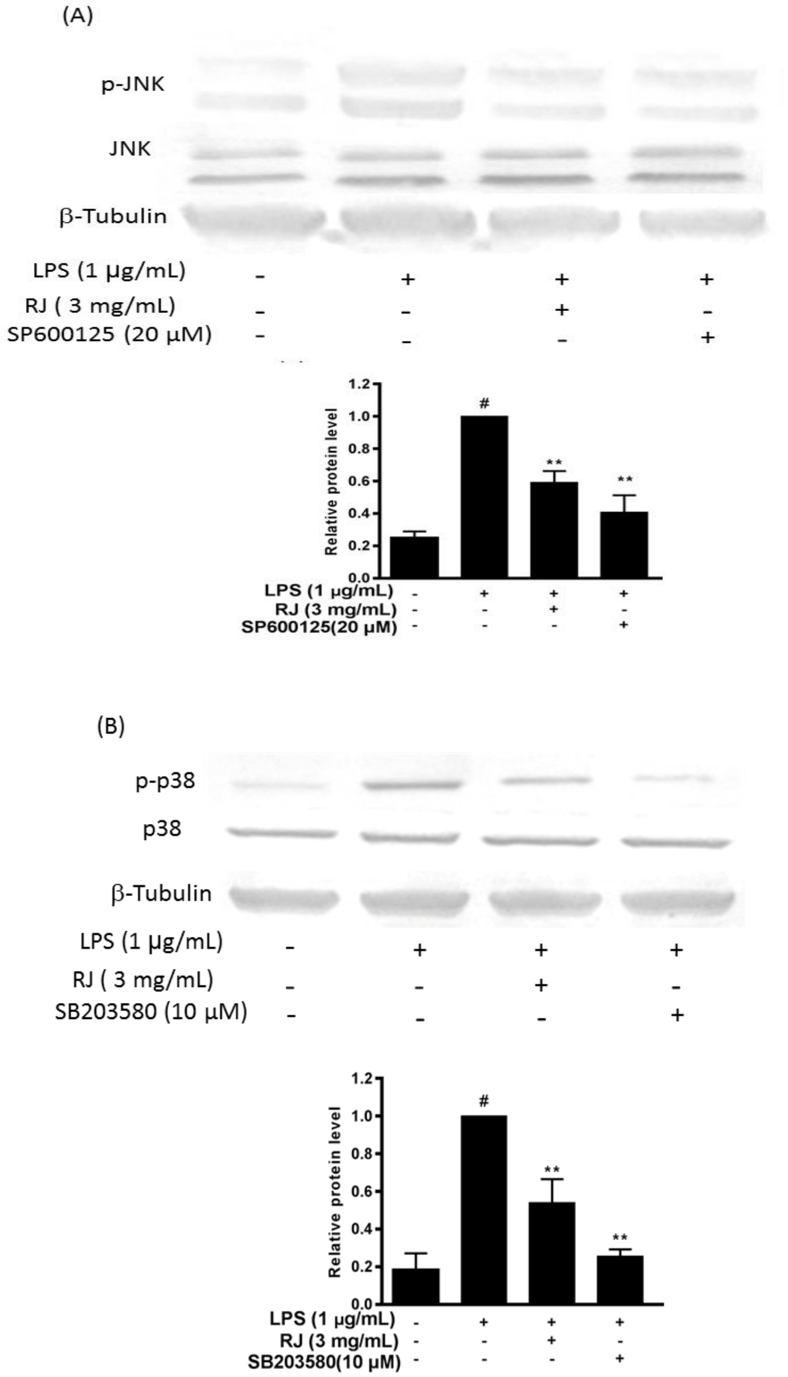

FIGURE 2: Effects of JNK inhibitor (SP600125) and p38 inhibitor (SB203580) on the phosphorylation of JNK and p38 in LPS-stimulated BV-2 cells. BV-2 cells were pretreated with or without RJ (3 mg/mL), SP600125 (20 μM) or SB203580 (10 μM) for 1 h, respectively, then treated with or without LPS for 45 min. The phosphorylated levels of JNK (A) and p38 (B) were detected by Western blot analysis. β-tubulin protein was used as an internal control. Data are presented as means ± SEM, and group differences were analyzed by one-way ANOVA with *post hoc* Tukey's test. $^{\#}P<0.05$ compared with untreated control group; $^{*}P<0.05$, $^{**}P<0.01$ compared with the group treated with LPS alone.
